# Risk Transmission Mechanism of Domestic Cluster Epidemic Caused by Overseas Imported Cases: Multiple Case Studies Based on Grounded Theory

**DOI:** 10.3390/ijerph191811810

**Published:** 2022-09-19

**Authors:** Xuefeng Li, Hui Jiang, Xiaoyu Liang

**Affiliations:** 1 School of Engineering Science, University of Chinese Academy of Sciences, Beijing 100049, China; 2 School of Public Policy and Management, University of Chinese Academy of Sciences, Beijing 100049, China

**Keywords:** domestic cluster epidemic, risk factors, risk transmission, grounded theory

## Abstract

The continued severity of the global epidemic situation has led to a rising risk of imported cases in China, and domestic cluster epidemic events caused by imported cases have occurred from time to time, repeatedly causing nation-wide disruption. To deeply explain this phenomenon, this study adopted the grounded theory method, using the 5·21 Guangzhou COVID-19 outbreak and 7·20 Nanjing COVID-19 outbreak as examples to study the risk transmission mechanism of domestic cluster epidemic caused by overseas imported cases. The study found that the risk factors for the phenomenon mainly include the following seven aspects: external protection, operations and supervision, international and domestic environment, contaminated objects, virus characteristics, management efficacy, and individual factors. These risk factors together constitute the “detonator”, “risk source”, “risk carrier,” and “risk amplifier” in the risk transmission process. In addition, this study also found that the transmission mechanism of domestic clusters caused by imported cases is a process of secondary risk amplification. The increase in risk carriers leads to a surge in secondary risks compared with the first, which leads to the outbreak of domestic clusters. Finally, based on the characteristics of the transmission mechanism and risk transmission components, this study provides some suggestions on risk mitigation for public departments to optimize China’s epidemic prevention policies.

## 1. Introduction

The COVID-19 outbreak in Wuhan in December 2019 was a surprise examination for the global emergency management system and governance capacities of all countries in the world. The WHO has listed it as a “Public Health Emergency of International Concern” that threatens the life and safety of people worldwide [1].

As of 29 December 2021, the global number of COVID-19 infections has exceeded 283 million, and the cumulative death toll has exceeded 5.42 million [2]. In the face of a previously unknown and unexpected epidemic, the Communist Party of China and the government have attached great importance to it and acted quickly; the national epidemic prevention and control campaign made significant strategic achievements. After March 2020, the spread of the domestic epidemic had basically been blocked, the CPC Central Committee firmly grasped the development and changes of the global epidemic situation, and timely action determined the prevention and control strategy of “preventing inbound cases and domestic resurgence” [3]. However, the epidemic situation in China is still facing sporadic outbreaks which have repeatedly caused disruption to the government and the people. Tracing the origins of these sporadic outbreaks led to findings that these domestic epidemics were caused by sources of overseas import, which is enough to show that the situation of “preventing inbound incursion” in China is severe.

In order to prevent imported cases from entering China through airports, ports, and borders, China formulated prevention guidelines for various places and has updated them in real time according to the epidemic situation.

However, for high-risk places such as international airports, ports, and borders with large passenger flows, dense population, and convenient transportation, the discovery of imported cases has become a normal phenomenon. If these “slip through the net cases” are not regulated in time, they could easily cause a domestic cluster epidemic. Faced with this current situation in China, this study attempts to answer the following questions:

- Within the process of individual imported cases turning into domestic cluster epidemics, what risk factors have not been effectively identified or seriously underestimated?
- What is the risk transmission mechanism in this process?
- How can these risks be effectively regulated to cope with the possible resurgence of domestic outbreaks in the future?

Therefore, based on the situation in China, this study selects representative events of overseas imported cases leading to domestic clusters, deeply refines the risk factors in this process, constructs the risk transmission model, and puts forward relevant suggestions on risk mitigation.

## 2. Literature Review

### 2.1. Study on Risk Factors of Novel Coronavirus Spread

Since 2020, a large number of scholars have conducted research on the risk factors for the spread of the novel coronavirus from the aspects of medicine, climate, transportation, occupation, etc. Among them, medical research was the earliest to be carried out and occupies the largest number of studies.

The novel coronavirus has been proven to be able to spread through aerosols [4], body contact and contaminated objects [5,6]. The risk of infection with the novel coronavirus or critical condition is affected by underlying diseases such as hypertension [7], diabetes, and dyslipidemia [8,9]. In addition, studies have shown that blood type, smoking habits, and the degree of obesity all affect the probability of infection. The immune system of the elderly usually weakens with age, so the severe disease rate and mortality rate in this group of the population are also higher than those of other groups [10].

The transmission rate of novel coronavirus is also affected by climatic and environmental factors. Studies have shown that the cold season accelerates the spread of the virus [11]. Precipitation, relative humidity, wind speed, air quality, and other factors also have an important impact on the transmission of the virus. Rendana’s study showed that the lower the wind speed, the more people were infected with COVID-19 [12]. Wang and other scholars conducted research on 100 cities in China that had the outbreak of COVID-19 and found that high temperatures and high relative humidity inhibits the spread of COVID-19 [13]. In addition, some scholars have also studied the effects of air pressure [14], precipitation, and solar radiation on the transmission of novel coronavirus [15,16].

Apart from the important influence of pathological factors and climatic environmental factors on the transmission of the novel coronavirus, the transportation networks can also accelerate the spread of the virus [17]. Large scale population mobility and convenient public transport can effectively promote the spread of the virus [18]. The connectivity and distance between the source of infection and the destination are also important factors affecting the rate and range of transmission of the virus [19]. Therefore, public transport has always been the focus of policy intervention aimed at curbing the current COVID-19 epidemic.

In addition, some scholars have studied the risk of infection from an occupation-centered perspective, especially doctors and front-line nurses. For example, the study by Dzinamarira and others concluded that lack of personal protective equipment, exposure to infected patients, work overload, poor infection control, and poor medical conditions are the main infection risk factors for health workers [20]. During the COVID-19 pandemic, “presenteeism” has a risk factor that occurs in all occupations, which is interpreted as “working despite symptoms of illness” [21]. The fundamental reason is that workers are afraid of losing their jobs and would rather take the risk of spreading the infection than miss work, which also explains why some workers still work after showing clinical symptoms of the new coronavirus.

### 2.2. Research on Risk Transmission

In 2005, scholars Ye and Deng defined risk transmission. They believed that the process of risk transmission within or between stakeholders through capital, technology, information, legal policies and other channels can be called risk transmission [22]. Niu believes that risk transmission is a dynamic process, including five basic elements: risk source, risk path, risk carrier, risk amplifier and risk consequence. The risk transmission process is affected by the butterfly effect, herd effect, and coupling effect, which further magnifies or intensifies the risk [23].

Hu believes that risk transmission happens in two ways: unitary and clustered. The mode of risk transmission from one carrier to another is called unitary transmission. This one-to-one transmission mode has the characteristics of slow speed, small scale, and easy traceability [24]. Clustered transmission is a multi-pathed conduction mode from the risk carrier as the center to the surrounding elements, which has the characteristics of one-to-many, many-to-many, fast speed, large scale, and difficult traceability. At the same time, Sun et al. found that risk is easily transmitted within network organizations, just as viruses spread among people. If people do not understand or control the risks, network organizations will potentially suffer a great negative impact. According to the different transmission directions, Sun et al. divided the network organization risk transmission into chain transmission, radiation transmission, and centralized transmission [25]. The process of risk transmission of the public health emergency caused by the new coronavirus is a typical centralized risk transmission, and the epidemic risk is transmitted by the risk source through multiple carriers.

At present, research related to risk transmission is mostly concentrated in the fields of supply chain, financial service, and transportation networks [26]. For instance, Feng et al. pointed out that risks in supply chains can be caused by the causal chain of risk factors, and spread and escalate through multiple paths, leading to changes in systemic risk [27]. Yang et al. used a novel risk spillover network method to study the dynamic evolution of the risk transmission relationship between various sectors of China’s financial market under the global COVID-19 epidemic outbreak. They examined the main sources and spillover paths of international financial risk transmission through extreme risk events such as the 4 “circuit breakers” in the U.S. stock market during the pandemic [28]. Through research on the transmission path of risks in the accident network, scholars such as Wang found that changing the transmission probabilities of non-critical paths cannot inhibit the transmission of risks in the accident network, and the critical paths containing important nodes should instead be controlled [29].

### 2.3. Research Review

Through a literature review in the fields of COVID-19 risk factors and risk transmission, it was found that the research on the risk factors of the novel coronavirus transmission focuses mostly on the fields of medicine, climate, transportation, etc., and the research results mostly analyze the COVID-19 virus transmission from a quantitative perspective. There is a lack of systematic identification and risk interaction research on the risk factors of COVID-19 epidemic events. In the early stage of the epidemic, it was very difficult to carry out quantitative research because of the lack of quantitative data. However, some valuable qualitative research results can help decision makers quickly grasp the crisis development trend and make correct decisions in time.

In addition, research on risk transmission is heavily concentrated in the fields of economics and finance. Emergency management research is mostly aimed at major accidents and emergencies of natural disasters. The mechanism of public health emergencies has obvious risk transmission effects, but the relevant research is relatively insufficient. Therefore, this study intends to take the perspective of management to study the risk transmission mechanism of domestic cluster epidemics caused by overseas imported cases, providing a reasonable interpretation framework and practical approach for optimizing China’s future epidemic prevention strategies, while enriching the research on the risk of public health emergencies in the category of emerging infectious diseases.

## 3. Research Design

### 3.1. Research Method

Grounded theory is a classical qualitative research strategy proposed by American scholars Glaser and Strauss. The method uses a practical observation approach to conduct systematic data collection, analysis, comparison, reflection, and transformation, so as to excavate, construct, or develop theories, and there are no theoretical assumptions prior to the research [30]. Grounded theory is very suitable for the problem of factor identification. It is suitable for researchers to find several factors that affect a certain problem from a large body of basic data and explore the causal relationship between variables, so as to construct the theory about the research problem [31].

**Figure 1:**
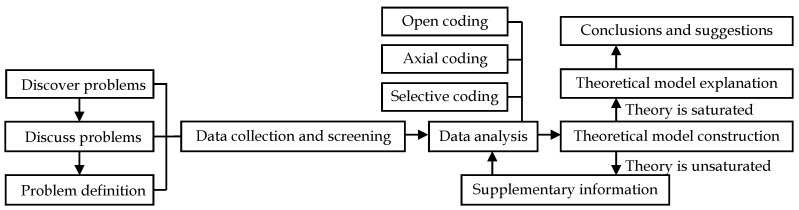
Flow chart of grounded theory research.

When using the grounded theory method to conduct case studies, we need to find scientific problems first, then discuss and define the problems, and collect and screen the data related to the problem. In the data analysis stage, researchers need to code text data, the coding process is divided into three steps: open coding, axial coding, and selective coding. After data analysis, a theoretical model is constructed according to the coding results, and it will be used to verify the theoretical saturation. If the theory reaches saturation, which means no new concept appears, the theoretical model can be further explained, give conclusions and suggestions; If the theory does not reach saturation, that is, new concepts appear, it is necessary to supplement information and modify the theoretical model until the theory reaches saturation before proceeding to the next step. The detailed flow chart is shown in Figure 1.

Based on the current situation in China, this study focuses on the phenomenon of “imported cases lead to domestic cluster epidemics”, using the grounded theory method to study the key risk factors and risk transmission effects in this process, then constructing a theoretical model to explain this phenomenon. The specific research process strictly follows the grounded theory research paradigm.

### 3.2. Sample Selection

After a comprehensive comparison of many domestic epidemic outbreaks, this study selected the 5·21 Guangzhou COVID-19 outbreak and 7·20 Nanjing COVID-19 outbreak as examples for this study. The specific reasons are as follows.

First, both the 5·21 Guangzhou COVID-19 outbreak and 7·20 Nanjing COVID-19 outbreak had a great impact on society, which aroused widespread concern and discussion. Scientific research has confirmed that the sources of the two epidemics are new variant strains imported from abroad. Therefore, the two cases are highly typical and representative.

Second, in terms of geographical location, Nanjing is located in the Yangtze River Delta in East China, Guangzhou is located in the Pearl River Delta in South China. Both of them are important transportation hubs in China. At the administrative level, the two cities are both provincial capitals, and their economic development level and degree of internationalization are at the forefront of the world, with similar international status. More importantly, the two cities are equipped with international airports with extremely high annual throughput. Compared with other high-risk places such as ports and borders, the social environment is more complex. The risk transmission rate is much faster when the risk of overseas imported cases occurs.

In addition, at the level of central deployment, even though the central government has been stricter in preventing and controlling the epidemic in municipalities directly under the central government and provincial capital cities than in other cities, there are still epidemic outbreaks caused by imported cases in the two cities, and the risk factors involved and their relationship need to be urgently explored.

Finally, in terms of data availability, after the epidemic rebounded, as the key areas of national epidemic prevention and control, a large number of media quickly tracked the progress of the epidemic, and key data such as various academic research results, official announcement, in-depth expert analysis, and stakeholder interviews can also be quickly obtained from the internet, providing sufficient data for this study.

### 3.3. Data Collection

This study used the 5·21 Guangzhou COVID-19 and 7·20 Nanjing COVID-19 outbreaks as examples and collected data from the internet. In order to ensure the timeliness, authority, and accuracy of the text data, the collection process focused on the authoritative information from the national, provincial, and municipal government official websites, and some academic papers, as shown in Table 1. After downloading the text data from the internet, we first read the text content roughly, assessed whether the text data was closely related to the research, and traced it back to the original report as far as possible according to the source of the text data. For forwarded reports, we tried to trace back to the original report according to the article title. For the same report forwarded by different media, only one copy of text data was retained. Through extensive collection, careful reading, and accurate screening, 140 documents were finally selected. Then 112 documents were randomly selected for analysis, and the remaining 28 documents were used for a theorical saturation test.

## 4. Case Rapid Review

### 4.1. 5·21 Guangzhou COVID-19 Outbreak Review

Guangzhou is the capital of Guangdong Province, an important central city and megacity in China, with a permanent population of 18.8706 million and a total area of 7434.40 square kilometers. It is a world-class city and an international comprehensive transportation hub. Guangzhou Baiyun International Airport is located in Baiyun District, Guangzhou city. It is one of the three national international aviation hubs, one of the Belt and Road Initiative, the Air Silk Road important international hub, and holds a core position at Guangdong-Hong Kong-Macao Greater Bay Area aviation hubs [32]. The airport’s routes cover more than 230 destinations, including nearly 90 international and regional destinations. In 2020, under the special circumstance of COVID-19 epidemic, Guangzhou Baiyun International Airport transported 43.768 million passengers, ranking first among airports in the world [33].

**Figure 2:**
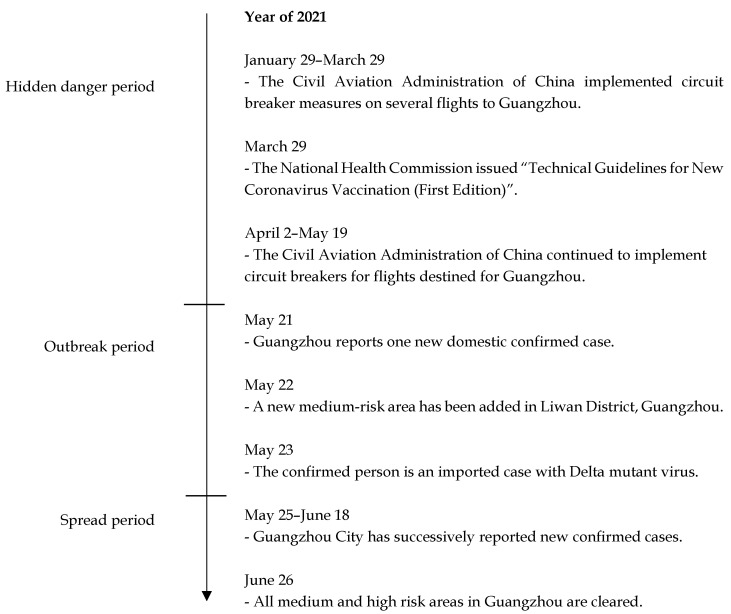
5·21 Guangzhou COVID-19 outbreak timeline.

During the epidemic period, Guangdong province accounted for 90% of the total number of people entering the country, bearing greater pressure on epidemic prevention and control than any other province. According to the development of the epidemic outbreak in Guangzhou on May 21, the epidemic can be divided into a hidden danger period, an outbreak period, and a spread period, as shown in Figure 2.

Hidden danger period: In order to further prevent and control the risk of imported cases from abroad, the Civil Aviation Administration of China implemented circuit breaker measures on inbound flights on 8 June 2020. For the same airline flight, if more than 5 passengers whose nucleic acid test results were positive after aircraft landing, the airline would be suspended for 2 weeks [34]. Since 2021, international flights destined for Guangzhou have repeatedly triggered the circuit breaker mechanism, laying a huge hidden danger for the 5·21 Guangzhou COVID-19 outbreak. On 29 March 2021, the National Health Commission issued the “Technical Guidelines for New Coronavirus Vaccination (First Edition)” to guide the vaccination of new coronavirus vaccines.

Outbreak period: On 21 May 2021, one domestic case was reported in Guangzhou. The next day, the Guangzhou Jinlong huixinge community was upgraded to a medium risk area. On May 23, virus tracing determined that the culprit was the Delta mutant virus. The mutant virus has the characteristics of short incubation period, fast transmission speed and high viral load. This is the first time that the mutant virus had been found to spread in communities in China. From May 22 to 24, although no new domestic cases were reported in Guangzhou, an outbreak of a cluster epidemic had already occurred.

Spread period: From May 25 to June 18, the epidemic situation in Guangzhou entered the spread period, with newly confirmed cases every day and 153 cases in total. On June 26, the medium and high risk areas in Guangzhou were cleared.

### 4.2. 7·20 Nanjing COVID-19 Outbreak Review

Nanjing is the capital of Jiangsu Province, the central city of East China, and one of China’s megacities. With a permanent population of 9.4234 million and an area of 6587.02 square kilometers, it is an important central city and comprehensive transportation hub in eastern China. Nanjing Lukou International Airport is located in Jiangning District, Nanjing. As the main trunk airport of China, the airport’s routes reach 78 major cities in China and 35 international and regional urban destinations. It is regarded as “an important hub of world-class airport clusters in the Yangtze River Delta”. In 2021, Nanjing Lukou Airport was the airport with the largest passenger throughput in Jiangsu Province, reaching 17.6069 million passengers.

**Figure 3:**
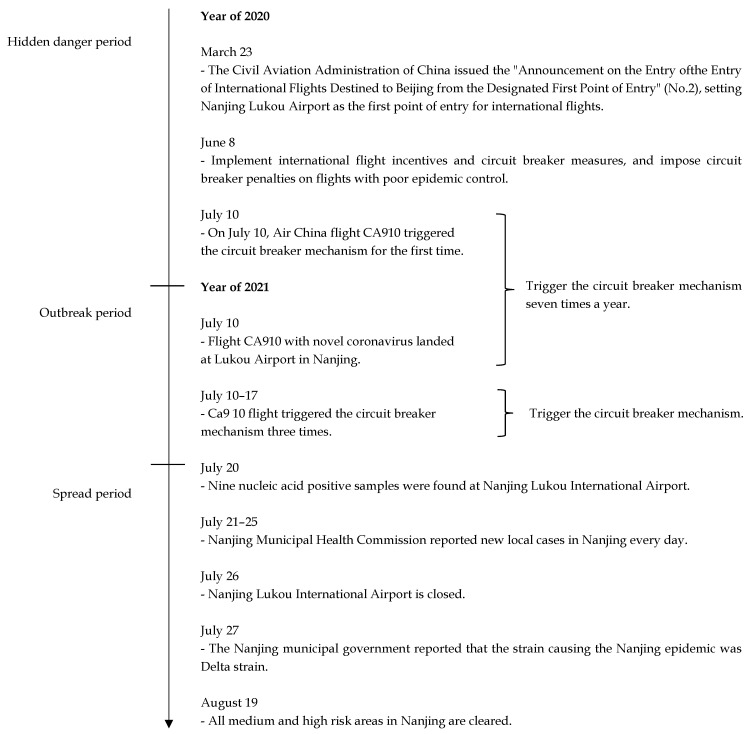
7·20 Nanjing COVID-19 outbreak timeline.

The cluster of COVID-19 outbreak in Nanjing started from 20 July 2021 at Nanjing Lukou International Airport, and ended on August 19 when the high-risk areas in Nanjing were cleared. The whole process can be also divided into three stages: hidden danger period, outbreak period and spread period, as shown in Figure 3.

Hidden danger period: On 23 March 2020, the Civil Aviation Administration, the Ministry of Foreign Affairs, the Health and Health Commission, the General Administration of Customs, and the Immigration Bureau jointly issued the “Announcement on the Entry of International Flights destined for Beijing from the Designated First Point of Entry (No. 2)”, making Nanjing the first point of entry for international passenger flights destined for Beijing. On 8 June 2020, the Civil Aviation Administration of China issued the “Notice of the Civil Aviation Administration on Adjusting International Passenger Flights” to implement circuit breakers and incentive measures for flights. On 10 July 2020, the Civil Aviation Administration issued a circuit breaker order for Air China flight CA910 (Moscow to Zhengzhou) for the first time to punish the flight for carrying more than 5 passengers with positive nucleic acid test results. In the following year, the flight frequently triggered the circuit breaker.

Outbreak Period: On 10 July 2021, flight CA910 landed at Nanjing Lukou International Airport. From July 10 to July 17, the circuit breaker mechanism was triggered three times, and the 7·20 Nanjing COVID-19 outbreak entered the outbreak period.

Spread period: On 20 July 2021, Nanjing Lukou International Airport detected nine positive nucleic acid test result, involving personnel in high-risk occupation such as airport ground service and cleaning, indicating that the epidemic in Nanjing had entered a spread period. On July 26, Nanjing Lukou International Airport was closed and a comprehensive disinfection was carried out. On July 27, the virus strain of the July 20 outbreak in Nanjing was confirmed as the Delta strain. On August 19, the medium and high-risk areas in Nanjing were cleared, marking the end of the Nanjing outbreak. However, by that time, the Nanjing epidemic outbreak had already spread to many provinces and cities in China.

## 5. Category Refinement and Model Construction

### 5.1. Open Coding

Open coding is a process of cutting up and giving concepts to letters, words, sentences, paragraphs, and whole texts in the original data. In this process, in addition to the systematic classification of the original data, it is also necessary to deeply mine the hidden concepts behind the materials in combination with the research situation, so as to realize the in-depth analysis of the research problem [35]. We first imported the text data collected from the two epidemic events into the NVivo 12 software. Second, we read each document carefully and numbered the risk factors that directly or indirectly related to formation of the epidemic situation. Finally, we classified the statements that represent the same risk factors in these texts, so as to extract the initial concept. At the open coding stage, a total of 21 initial concepts were analyzed. Then, these initial concepts were classified again, and 20 initial categories were obtained.

### 5.2. Axial Coding

Axial coding is a process of comparing and analyzing the categories summarized by open coding again, so as to cluster the main categories. After further refining the 20 initial categories obtained in the open coding stage, 14 main categories were obtained.

### 5.3. Selective Coding

Selective coding is the process of further refining the main category to obtain the core category, and summarizing a higher-level “story line” to summarize the whole logic. This study reanalyzes and clusters the 14 main categories extracted from the selective coding to obtain the 7 core categories: external protection, operations and supervision, international and domestic environment, contaminated objects, virus characteristics, management efficacy, and individual factors.

The example of coding process using by grounded theory is shown in Table 2.

The complete coding results are shown in Table 3.

### 5.4. Model of Risk Transmission Mechanism of Domestic Cluster Epidemic Caused by Imported Cases

**Figure 4:**
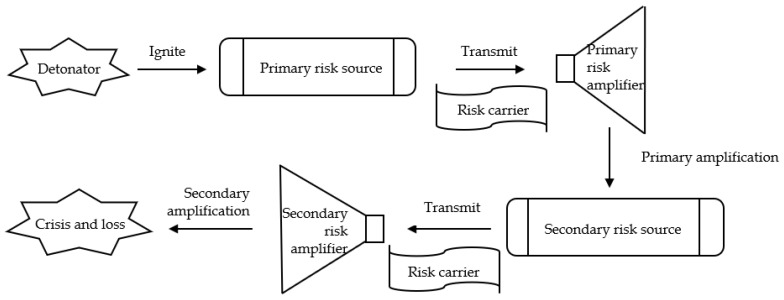
Risk transmission schematic diagram of the domestic cluster epidemic caused by overseas imported cases.

According to the risk transmission mechanism and the grounded theory coding results, the risk transmission schematic diagram of the domestic cluster epidemic caused by overseas imported cases is constructed. As shown in Figure 4, the model mainly contains four elements, which are detonator, risk source, risk carrier, and amplifier.

In the process of risk transmission, the detonator is usually composed of the system and social environment, and the explosion of the detonator will cause the formation of the primary risk source. If the primary risk source cannot be controlled or resolved in a timely and effective manner, the risk will quickly spread outward with the help of the risk carrier.

Moreover, in the process of risk transmission, the interaction of various risk factors may amplify the risk carried by the risk carrier due to the coupling effect, butterfly effect, broken window effect and other effects, and it would become easier to generate a secondary risk source with the help of the unsafe behavior of people or the unsafe state of objects, and cause the risk carrier to produce a wider range of transmission. If the risk transmission is not interrupted in time, the risk will be amplified again through the interaction of various risk factors, leading to a domestic epidemic outbreak.

**Figure 5:**
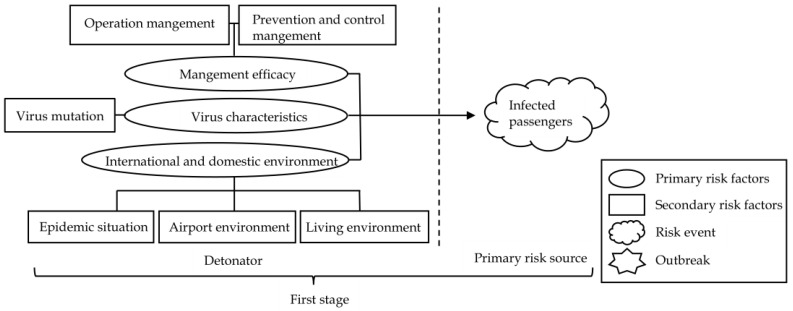
First stage of risk transmission process.

**Figure 6:**
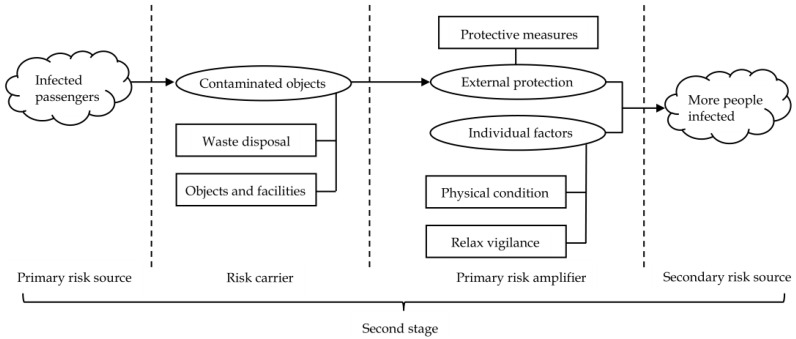
Second stage of risk transmission process.

**Figure 7:**
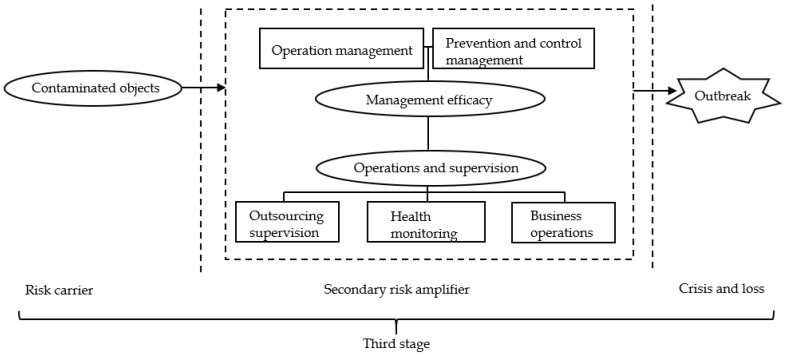
Third stage of risk transmission process.

According to the categories obtained from the grounded theory, combined with Figure 4, the risk transmission mechanism model of the domestic cluster epidemic caused by overseas imported cases was constructed. To better present the whole risk transmission process, the model was divided into three parts, each part representing one stage based on the risk transmission mechanism, as shown in Figure 5, Figure 6 and Figure 7.

### 5.5. Theorical Saturation Test

In order to test the theoretical saturation of the risk transmission model, this study used the NVivo 12 software to conduct open coding, spindle coding, and selective coding for the remaining 28 data samples again, according to the grounded theory research steps. After repeating the coding steps, no new concepts, categories, and relationships were found, indicating that the theoretical model passed the theoretical saturation test.

## 6. Discussion

The risk transmission mode of the domestic cluster epidemic caused by imported cases from abroad is different from that in the supply chain, finance, transportation, and other fields. The risk transmission mode is a process of secondary amplification. Due to the increase in risk carriers, the magnification of the second amplification is significantly higher than that of the first amplification, which directly leads to the outbreak of the domestic cluster.

At the first stage, as shown in Figure 5, “virus characteristics”, “international and domestic environment”, and “management efficacy” in the social system together constitute the detonator, which can be detonated at any time. The mutated virus has strong transmission ability because of its own pathogenic characteristics.

Important transportation hubs such as airports, ports, borders, and other high-risk places are the first checkpoints for foreign personnel to enter the country. These places have complex personnel and strong population mobility. If there is a lack in daily operations and management, or an increase in the number of people during special periods such as during the Spring Festival and summer vacation, the risk of cluster outbreaks would sharply rise.

Under the high-risk background of a global pandemic, healthy people outside the country may actively or passively come into contact with the virus and detonate the “detonator”, resulting in viral infection and leading them to become a “primary risk source”.

At the second stage shown in Figure 6, if the infected passengers are not identified, controlled, and treated in a timely and accurate manner, and enter into China with the help of international transportation, the virus would be brought into China. Passengers carrying mutated viruses may also come into contact with objects, facilities, air, etc., in transportation facilities, resulting in contaminated surfaces, which leads to these objects becoming “risk carriers”.

After arriving from international flights, staff in high-risk occupations must immediately conduct comprehensive disinfection and quarantine. Efficient and thorough disinfection can effectively interrupt risk transmission and significantly reduce the risk of cluster outbreaks. If the virus is not completely eliminated due to incomplete disinfection and inadequate supervision in the process, the risk will continue to be transmitted through the risk carriers. In this process, the “external protection” of people in high-risk occupations, and “individual factors” constitute a “barrier” against the virus.

“External protection” refers to the protective clothing, masks, gloves, and other protective equipment worn by the staff according to the provisions of the epidemic prevention guidelines. If the staff do not have sufficient protective measures, they may be infected in the virus contaminated environment and quickly infect others through the living area. Individual factors such as the status of their own physical condition and the level of vigilance, will greatly enlarge or reduce the system risk. If the barrier formed by these two factors is solid, the risk transmission may be interrupted; if the barrier is weak, it will become a “primary risk amplifier”, magnify the risk and accelerate the risk transmission with the help of more risk carriers.

At the third stage as shown in Figure 7, after being amplified by the primary risk amplifier, the risk will be transmitted with the help of more “risk carriers”. At this time, the system risk will face a new “barrier”, which is the last line of defense against a domestic cluster. The line consists of two aspects: “operations and supervision” and “management efficacy”.

First, the risks in “operations and supervision” mainly focus on “health monitoring”, “outsourcing supervision”, and “business operations”. In terms of “health monitoring”, the frequency and accuracy of nucleic acid tests are very crucial. Low frequency and low accuracy of nucleic acid tests will lead to infected persons not being timely controlled, which will accelerate the spread of risk.

In terms of “outsourcing supervision”, the risk is that after the airport outsources its business to a third-party company, the airport does not strictly supervise the outsourcing company, or both the airport and the outsourcing company think that the management right belongs to the other party, resulting in a management gap.

When the staff of the outsourcing company are performing business operations, the standardization of operation steps will also affect the spread of the virus. For example, after an international flight enters the country, the cleaners need to disinfect the items and the environment that may be contaminated by the virus. If the cleaner’s business operation is not standardized, the system risk will be further transmitted.

In the daily operation of the airport, the operation of international and domestic flights should be separated to avoid the spread of the epidemic due to operational management problems. In addition, poor prevention and control management will also aggravate the risk of domestic clusters. When positive samples are found in high-risk locations, centralized management, and control of personnel in high-risk positions must be carried out immediately, and strong tracking measures must be taken. If the control of high-risk personnel is delayed, these above risk factors will become a “secondary risk amplifier”, surging the systemic risk again on the first amplification, leading to the epidemic outbreak.

The interaction between the above-mentioned risk factors in the process of risk transmission can cause a system risk to break through layers of barriers and pass through the detonators, risk sources, risk amplifiers, and other important components, leading to the outbreak of domestic clusters.

## 7. Risk Mitigation and Suggestions

Domestic epidemics caused by overseas imported cases is formed by the joint action of a series of risk factors, which constitute the risk transmission components such as “detonator”, “risk source”, “risk carrier”, and “risk amplifier”, each component is closely related to each other. In the risk transmission process, the system risk can be reduced by regulation of any component or relationship. However, different regulatory measures will lead to different regulatory effects. For domestic clusters caused by overseas imported cases, specific regulatory recommendations are as follows.

### 7.1. Establish a Whole-Process Risk Response Mechanism to Prevent and Resolve Risks in a Timely Manner

This regulatory recommendation addresses the entire risk transmission process. From the model established in Figure 5, Figure 6 and Figure 7, it can be seen that the domestic cluster epidemic caused by imported cases is the result of the combined effect of various risk factors. These risk factors are closely linked, and exist independently or throughout the hidden danger period, outbreak period, and spread period. For the risk regulation of domestic epidemics caused by imported cases from abroad, the whole process of hidden danger period, outbreak period, and spread period should be considered, and the regulation should be carried out from the perspective of the whole risk transmission process. Therefore, it is necessary to establish a comprehensive and systematic whole-process risk mechanism that integrates risk monitoring, assessment, tracking control, and systematic resolution to reduce the risk of domestic clusters caused by imported cases. In the risk monitoring and assessment mechanism, high-risk places can conduct risk monitoring and preliminary assessments according to the characteristics of the inbound population and means of transport (such as passengers in countries with very serious epidemics or frequently suspended flights), so as to improve the risk prevention capacity.

At the same time, it is necessary to establish a systematic in-process evaluation and tracking control mechanism for suspected epidemic events. For example, after positive nucleic acid test samples are detected, it is necessary to fully evaluate what control measures need to be taken, determine the necessity of comprehensive closed-looped management, and quickly track and control all high-risk people in the local site, so as to improve the risk control ability in the whole process.

Finally, a systematic risk resolution mechanism should be established to run through the whole risk transmission process. For the risks that may occur at any stage of the risk transmission process, a special countermeasure library is needed to facilitate the decision-makers to select reasonable countermeasures according to the actual situation and quickly resolve the risks in the process of risk monitoring, assessment, and tracking.

### 7.2. Establish a Learning Mechanism for Dynamic Epidemic Prevention Measures Based on the Core Business and Environmental Characteristics

This regulatory recommendation is aimed at primary risk amplifiers. High-risk locations such as international airports, ports, and borders are the first checkpoints for China’s overseas imports. China has formulated detailed epidemic prevention regulations for epidemic prevention and control measures in these high-risk locations to guide the work of personnel in high-risk occupations. As the first barrier against viruses for high-risk personnel, the strength of external protection is directly related to the risk of infection. In the 7·20 Nanjing COVID-19 outbreak, the “Technical guidelines for epidemic prevention and control of transport airlines and airports (Seventh Edition)” did not set mandatory requirements for cleaners to wear protective clothing, which possibly increased their exposure to the risk environment.

For high-risk locations, various epidemic prevention guidelines and regulations issued by the relevant government departments should be used as the “lower limit” of the extent of the epidemic prevention measures. Then, based on risk level of the actual situation, formulate suitable epidemic prevention measures to regulate the system risk. For example, most provincial capital cities and municipalities have international airports which usually have extremely high passenger flow, which also leads to higher magnification of the “secondary risk amplification” and faster risk transmission. Therefore, the management measures for personnel in high-risk positions need to specify stricter epidemic prevention measures than domestic airports. In addition, as the epidemic situation in China has been dynamic, many epidemic prevention policies are often formulated based on “empirical learning” after the rebound of the epidemic outbreak, which comes at a high social cost. Therefore, for different risk areas, it is suggested to establish a dynamic learning mechanism on the basis of national policy guidance, regularly review the deficiencies in management, and make up for them before the crisis occurs, so as to “nip it in the bud”.

### 7.3. Carefully Select Outsourcing Business and Establish a Regulatory System

This regulatory suggestion is mainly aimed at detonators and secondary risk amplifiers. Business outsourcing is an important part of the modern service industry and has been applied to various industries. Many companies in high-risk places will outsource their non-core businesses to third-party companies, and the management organization will change from direct management type to operation and management type, so that they can focus more on the development of their main business, and achieve the purpose of reducing costs, improving operation efficiency, and enhancing their core competitiveness. However, any type of business outsourcing is accompanied by risks of security, market environment, performance quality, project management and others, which directly affects the normal operation of the management system. Therefore, when a high-risk location develops its own business, it first needs to conduct a systematic and overall risk assessment according to its own development status, current management status, and future development planning, then choose whether to operate directly or choose business outsourcing.

Secondly, the epidemic risk control in high-risk places is extremely important for the whole epidemic prevention and control. Even if business outsourcing is selected, it must be strictly screened according to the importance of business categories. The higher the risk, such as international flight cleaning, waste transportation, and other businesses, the more cautions that must be taken. Decision makers must fully study and judge the overall risk before deciding whether to adopt the outsourcing. Finally, even if business outsourcing is selected, it is also necessary to clarify the responsibility boundary between the owner and the partner. It is best to formulate a complete management system separately according to the actual situation, and form an institutional system with clear standards, clear responsibilities, and a standardized operation to supervise the business quality.

### 7.4. Establish a Whole-Process Risk Tracking Mechanism to Quickly and Accurately Track High-Risk Personnel

This regulation proposal is mainly aimed at the key links between various risk transmission elements. From the model established in Figure 5, Figure 6 and Figure 7, it can be seen that after the effects of the “primary risk amplifier” and “secondary risk amplifier”, more people would be exposed to the risk, and the people infected by the virus would become a new risk source, and spread to all parts of the country through convenient transportation, which further aggravates the risk of an epidemic outbreak. Therefore, strong tracking and control of these “new risk sources” and their contacts who have crossed time and space will directly affect the progress of the epidemic situation. However, at present, China’s powerful tracking means for these high-risk groups are still mostly through telephone contact, internet notice and government announcement. This method is rough and inefficient and easily causes omissions. From the whole process of epidemic prevention and control in China, it can be seen that using big data to carry out epidemic prevention activities can significantly improve the epidemic responsibility.

Using “Health QR codes” and “Travel codes”, relevant departments can establish a more accurate and complete risk tracking mechanism with the help of commonly used user interfaces. This mechanism can not only carry out daily dynamic risk monitoring for the working population in high-risk places, but also quickly notify the people who have crossed time and space with these high-risk populations in case of a local epidemic rebound, so as to achieve more rapid and accurate tracking and timely control of the generation, transmission, and spread of epidemic risk.

## 8. Conclusions

In the face of the severe global epidemic situation, imported cases from abroad have repeatedly lead to domestic epidemics in China, thereby causing a social impact. This research studied the risk transmission mechanism of this phenomenon. To identify the risk factors related to this phenomenon, this study selected the 5·21 Guangzhou epidemic outbreak and the 7·20 Nanjing epidemic outbreak in China as case samples, then used the grounded theory method to carry out in-depth research:

(1) By reviewing the two cases according to the timeline, we studied the risk factors of the domestic cluster epidemic caused by overseas imported cases, including 7 primary risk factors and 14 secondary risk factors. Among them, the primary risk factors are mainly concentrated into seven aspects: external protection, operations and supervision, international and domestic environment, contaminated objects, virus characteristics, management efficacy, and individual factors.
(2) This study found that, unlike the risk transmission in other fields, the risk transmission mechanism of the domestic cluster epidemic caused by overseas imported cases is a process of secondary risk amplification.

This study believes that in the risk transmission process, overseas healthy people contracted the virus before international travel, which became a source of risk, and then entered China with the help of international transportation. In this process, the goods and environment that have come into contact with a risk source have become risk carriers, and the system risk has been initially amplified, but it is still within the controllable range. However, if these risk carriers are not cleared in time, they will infect more healthy people. Infected people will become a secondary risk source and continue to amplify the risk. At this time, the system risk will surge out of control, resulting in a domestic epidemic. In view of the risk transmission mechanism of such events, this study gives suggestions on risk mitigation, which provides a valuable reference for the government to formulate better epidemic prevention policies in the future.

Compared with other studies, this research studies the whole process of risk transmission from the perspective of management, and discusses the correlation between various risk factors, which is conducive to decision makers to make correct decisions quickly when dealing with such events. In addition, this study builds a risk transmission model for such events based on the risk transmission theory, further expanding the application of the risk transmission theory in the field of public health emergencies.

Although this study answers the questions raised in the introduction, there are still some limitations. First, the data sources of this study are all collected from the internet, lack first-hand data sources such as interviews or questionnaires. Second, this study selects two China’s cases as samples, and the applicability of the research conclusions in the world needs to be verified by the cases of other countries. Third, the risk places involved in the case samples are all airports, but whether the risk transmission mechanism of high-risk places such as ports and borders is the same as the conclusion of this study needs to be further verified by collecting relevant data. Therefore, on the basis of this study, future research can add data sources such as interviews and questionnaires, and select cases from different countries and places for research to make up for the limitations of this study.

## Figures and Tables

**Table 1:** List of data sources.

	Data	Government Official Website	Local Media Representatives	National Party Media	Authoritative We Media	News Interview
Outbreak						
5·21 Guangzhou COVID-19 Outbreak		Guangzhou Municipal people’s Government official website	Guangzhou Bendibao, etc.	People’s Daily, Healthy Times, etc.	Dingxiangyuan	Airport staff interviews, expert interviews, Epidemic Prevention and Control Media Interviews, etc.
		Guangdong Provincial People’s Government official website				
		Guangzhou Health Commission official website				
7·20 Nanjing COVID-19 Outbreak		Nanjing Municipal people’s Government official website	Nanjing Bendibao, etc.			
		Jiangsu Provincial People’s Government official website				
		Nanjing Health Commission official website				

**Table 2:** Example of coding process in grounded theory.

Typical Evidence	Initial Conceptualization	Open Coding	Axial Coding	Selective Coding	Source
After investigation, it was found that these cleaning employees participated in the cabin cleaning of flight ca910. After the work was finished, because of the non-standard protective wash out, it may cause infection of individual cleaning personnel, and then spread among cleaning employees.	Nonstandard protective clothing	Inadequate daily protection	Protective measures	External protection	https://m.gmw.cn/2021-07/30/content_1302444088.htm, accessed on 1 August 2021
In the past, the disinfection of the aircraft cabin was to spray all the cabin and close the cabin door for several hours before someone went in to clean it. Now, this process involves spraying the cabin immediately after the passengers leave, and then going in and cleaning it without waiting at all.	Nonstandard disinfection operations	Nonstandard operations	Business operations	Operations and supervision	http://www.inewsweek.cn/society/2021-08-09/13415.shtml, accessed on 10 August 2021
Statistics on COVID-19 released by Johns Hopkins University in the United States show that as of 15:21 a.m. ET on August 4, the cumulative number of COVID-19 confirmed cases in the world exceeded 200 million, and the cumulative number of deaths was 4,252,873.	Overseas cases continue to increase	Severe overseas epidemic situation	Epidemic situation	International and domestic environment	http://www.xinhuanet.com/photo/2021-08/05/c_1127732127.htm, accessed on 5 August 2021
When cleaning personnel boarded the plane, they were exposed to the surfaces contaminated by passengers carrying virus.	Contaminated objects in passenger cabin	Contaminated airport facilities	Objects and facilities	Contaminated Objects	http://www.nhc.gov.cn/xcs/fkdt/202107/7d6894a8006d4cc891e25a4ced5aa27c.shtml, accessed on 3 August 2021
Lina Tao believes that the reason why this wave of epidemic spreads so quickly is mainly because the delta strain has a fast reproduction speed and strong transmission power.	Easy transmission of mutated virus	Increased infectivity of mutated virus	Virus mutation	Virus characteristics	http://www.inewsweek.cn/society/2021-08-06/13403.shtml, accessed on 8 August 2021
The airport also has the problem of unprofessional management, which changes the original separate operation of international flights and domestic flights into unified mixed operation, resulting in the inflow of overseas epidemic and the spread of epidemic.	Mixed operation of international and domestic flights	Mixed flight operation	Operation management	Management efficacy	https://www.ccdi.gov.cn/pln/202107/t20210728_142075.html, accessed on 10 August 2021
The long-term stable operation has made the airport employees relax their vigilance and be careless.	Mental relax and slack	Vigilance decrease	Relax vigilance	Individual factors	http://www.nhc.gov.cn/xcs/fkdt/202108/5dbf709cea7a4bcfb7beb5797802ad18.shtml, accessed on 12 August 2021

**Table 3:** Complete coding results.

No.	Initial Conceptualization	Open Coding	Axial Coding	Selective Coding
1	Imperfect protection provisions	Imperfect protection guide	Protective measures	External protection
2	Nonstandard protective clothing	Inadequate daily protection		
3	Outsourcing supervision of cleaning business	Outsourcing supervision of airport business	Outsourcing supervision	Operations and supervision
4	Low nucleic acid tests frequency	Low monitoring frequency	Health monitoring	
5	Nonstandard disinfection operations	Nonstandard operations	Business operations	
6	Overseas cases continue to increase	Severe overseas epidemic situation	Epidemic situation	International and domestic environment
7	High overseas import risk	Domestic epidemic threat		
8	Important geographical location	High passenger flow at transportation hub	Airport environment	
9	High airport throughput			
10	Inbound flights frequently suspended	High risk international flights		
11	Increased passenger flow in summer	People flow surge in special time		
12	People gathering in public space	Densely populated living place	Living enviornment	
13	Mixed international and domestic garbage cleaning and transportation	Garbage cleaning and transportation	Waste disposal	Contaminated objects
14	Contaminated objects in passenger cabin	Contaminated airport facilities	Objects and facilities	
15	Easy transmission of mutated virus	Increased infectivity of mutated virus	Virus mutation	Virus characteristics
16	Increased viral load of mutated virus	High viral load of mutated virus		
17	Mixed operation of international and domestic flights	Mixed flight operation	Operation management	Management efficacy
18	Mixed work of international and domestic flight staff	Mixed work of high-risk staff		
19	Poor controlled of high risk people	Poor prevention and control management	Prevention and control management	
20	Underlying disease	Physical function decrease	Physical condition	Individual factors
21	Mental relax and slack	Vigilance decrease	Relax vigilance	

## Data Availability

The data sets of the study are available from the corresponding author on reasonable request.

## References

[1] WHO Director-General’s Statement on IHR Emergency Committee on Novel Coronavirus (2019-nCoV). https://www.who.int/director-general/speeches/detail/who-director-general-s-statement-on-ihr-emergency-committee-on-novel-coronavirus-(2019-ncov).

[2] Real Time Dynamics of Novel Coronavirus Epidemic. https://ncov.dxy.cn/ncovh5/view/pneumonia.

[3] The State Council Information Office of the People’s Republic of China (2020). White paper-Fighting COVID-19: China in Action.

[4] Van Doremalen N., Bushmaker T., Morris D.H., Holbrook M.G., Gamble A., Williamson B.N., Tamin A., Har-court J.L., Thornburg N.J., Gerber S.I. (2020). Aerosol and surface stability of SARS-CoV-2 as compared with SARS-CoV-1. N. Engl. J. Med..

[5] Huang C., Wang Y., Li X., Ren L., Zhao J., Hu Y., Zhang L., Fan G., Xu J., Gu X. (2020). Clinical features of patients infected with 2019 novel coronavirus in Wuhan, China. Lancet.

[6] Lu C., Liu X., Jia Z. (2020). 2019-NCoV transmission through the ocular surface must not be ignored. Lancet.

[7] Li X., Xu S., Yu M., Wang K., Tao Y., Zhou Y., Shi J., Zhou M., Wu B., Yang Z. (2020). Risk factors for severity and mortality in adult COVID-19 inpatients in Wuhan. J. Allergy Clin. Immun..

[8] Guo L., Shi Z., Zhang Y., Wang C., Moreira N.C.D.V., Zuo H., Hussain A. (2020). Comorbid diabetes and the risk of disease severity or death among 8807 COVID-19 patients in China: A meta-analysis. Diabetes Res. Clin. Pract..

[9] Atmosudigdo I.S., Lim M.A., Radi B., Henrina J., Yonas E., Vania R., Pranata R. (2021). Dyslipidemia increases the risk of severe COVID-19: A systematic review, meta-analysis, and meta-regression. Clin. Med. Insights Endocrinol. Diabetes.

[10] Lithander F.E., Neumann S., Tenison E., Lloyd K., Welsh T.J., Rodrigues J.C., Higgins J.P., Scourfield L., Christensen H., Haunton V.J. (2020). COVID-19 in older people: A rapid clinical review. Age. Ageing.

[11] Liu X., Huang J., Li C., Zhao Y., Wang D., Huang Z., Yang K. (2021). The role of seasonality in the spread of COVID-19 pandemic. Environ. Res..

[12] Rendana M. (2020). Impact of the wind conditions on COVID-19 pandemic: A new insight for direction of the spread of the virus. Urban Clim..

[13] Wang J., Tang K., Feng K., Lin X., Lv W., Chen K., Wang F. (2021). Impact of temperature and relative humidity on the transmission of COVID-19: A modelling study in China and the United States. BMJ Open.

[14] Zhu Y., Xie J., Huang F., Cao L. (2020). The mediating effect of air quality on the association between human mobility and COVID-19 infection in China. Environ. Res..

[15] Mcclymont H., Hu W. (2021). Weather variability and COVID-19 transmission: A review of recent research. Int. J. Environ. Res. Public Health.

[16] Yao Y., Pan J., Liu Z., Meng X., Wang W., Kan H., Wang W. (2020). No association of COVID-19 transmission with temperature or UV radiation in Chinese cities. Eur. Respir. J..

[17] Browne A., St-Onge Ahmad S., Beck C.R., Nguyen-Van-Tam J.S. (2016). The roles of transportation and transportation hubs in the propagation of influenza and coronaviruses: A systematic review. J. Travel Med..

[18] Kraemer M.U., Yang C., Gutierrez B., Wu C., Klein B., Pigott D.M., Group O.C.D.W., Du Plessis L., Faria N.R., Li R. (2020). The effect of human mobility and control measures on the COVID-19 epidemic in China. Science.

[19] Zheng R., Xu Y., Wang W., Ning G., Bi Y. (2020). Spatial transmission of COVID-19 via public and private transportation in China. Travel Med. Infect. Dis..

[20] Dzinamarira T., Nkambule S.J., Hlongwa M., Mhango M., Iradukunda P.G., Chitungo I., Dzobo M., Map-ingure M.P., Chingombe I., Mashora M. (2022). Risk factors for COVID-19 infection among healthcare workers. A first report from a living systematic review and meta-Analysis. Saf. Health Work.

[21] Kinman G., Grant C. (2021). Presenteeism during the COVID-19 pandemic: Risks and solutions. Occup. Med..

[22] Ye H.Y., Deng M.R. (2005). Six ways and characteristics of enterprise risk transmission. Mod. Manag..

[23] Niu X.Y. (2014). Research on cost risk transmission mechanism of engineering project construction stage. Proj. Manag. Tech..

[24] Hu X.M. (2020). Formation mechanism and transmission mechanism of major social risks. Governance.

[25] Sun G.Q., Qiu Y.X., Li J.M. (2015). Research on the dynamic evolution pathes of risks transmission for network organization. Chin. J. Manage. Sci..

[26] Fu Z., Hu Z.A., Qiu Z.Q. (2021). Research on the risk transmission mechanism of multimodal transportation networks under the impact of the COVID-19 outbreak. J. Saf. Environ..

[27] Feng N., Wang H.J., Li M. (2014). A security risk analysis model for information systems: Causal relationships of risk factors and vulnerability propagation analysis. Inform. Sci..

[28] Yang Z.H., Chen Y.T., Zhang P.M. (2020). Macroeconomic shock, financial risk transmission and governance response to major public emergencies. J. Manag. World.

[29] Wang S., Zhan R.J., Ma Y.Z. (2016). Emulational analysis of risk transfer route of complex accident system. China Saf. Sci. J..

[30] Chen X.M. (1999). Thoughts and methods of grounded theory. Educ. Res. Exp..

[31] Jia Z.M. (2015). An Overview on Grounded Theory and its Applications in Public Administration: Methods and practical issues. Chin. Public Adm..

[32] Airport Overview. https://www.gbiac.net/byairport-web/menu/index?urlKey=airport-basic-facts_en.

[33] Guangzhou Baiyun International Airport to Be, No. 1 in the World in Terms of Passenger Throughput by 2020. http://www.sasac.gov.cn/n2588035/n2588330/n2588365/c16741622/content.html.

[34] Notice of the Civil Aviation Administration on Adjusting International Passenger Flights. http://www.caac.gov.cn/XXGK/XXGK/TZTG/202006/t20200604_202928.html.

[35] Juliet M.C., Anselm L.S. (2015). Foundation of Qualitative Research: The Procedure and Method of Forming a Grounded Theory.

